# Assessment Accuracy of 2D vs. 3D Imaging for Custom-Made Acetabular Implants in Revision Hip Arthroplasty

**DOI:** 10.3390/jpm14080808

**Published:** 2024-07-30

**Authors:** Timo Albert Nees, Christian Thomas Mueller, Moritz Maximilian Innmann, David Maximilian Spranz, Fabian Westhauser, Tobias Renkawitz, Tobias Reiner, Tilman Walker

**Affiliations:** Department of Orthopaedics, Heidelberg University Hospital, Schlierbacher Landstraße 200a, 69118 Heidelberg, Germany

**Keywords:** total hip arthroplasty, revision, custom-made implants, acetabular defects, Paprosky III, personalized medicine, additive manufacturing, 3D printing, CT, radiographs

## Abstract

Revision total hip arthroplasty (rTHA) presents significant challenges, particularly in patients with severe acetabular bone defects. Traditional treatment options often fall short, leading to the emergence of custom-made 3D-printed acetabular implants. Accurate assessment of implant positioning is crucial for ensuring optimal postoperative outcomes and for providing feedback to the surgical team. This single-center, retrospective cohort study evaluates the accuracy of standard 2D radiographs versus 3D CT scans in assessing the positioning of these implants, aiming to determine if 2D imaging could serve as a viable alternative for the postoperative evaluation. We analyzed the implant positions of seven rTHA patients with severe acetabular defects (Paprosky ≥ Type IIIA) using an alignment technique that integrates postoperative 2D radiographs with preoperative CT plans. Two independent investigators, one inexperienced and one experienced, measured the positioning accuracy with both imaging modalities. Measurements included translational shifts from the preoperatively templated implant position in the craniocaudal (CC), lateromedial (LM), and ventrodorsal (VD) directions, as well as rotational differences in anteversion (AV) and inclination (INCL). The study demonstrated that 2D radiographs, when aligned with preoperative CT data, could accurately assess implant positions with precision nearly comparable to that of 3D CT scans. Observed deviations were 1.4 mm and 2.7 mm in CC and LM directions, respectively, and 3.6° in AV and 0.7° in INCL using 2D imaging, all within clinically acceptable ranges. For 3D CT assessments, mean interobserver variability was up to 0.9 mm for translational shifts and 1.4° for rotation, while for 2D alignment, observer differences were 1.4 mm and 3.2° for translation and rotation, respectively. Comparative analysis of mean results from both investigators, across all dimensions (CC, LM, AV, and INCL) for 2D and 3D matching, showed no significant differences. In conclusion, conventional anteroposterior 2D radiographs of the pelvis can sufficiently determine the positioning of custom-made acetabular implants in rTHA. This suggests that 2D radiography is a viable alternative to 3D CT scans, potentially enhancing the implementation and quality control of advanced implant technologies.

## 1. Introduction

Primary total hip arthroplasty (THA) is recognized as a highly successful surgical intervention [1]. With the aging population and the increasing prevalence of joint diseases, the demand for THA [2] and its revision (rTHA) [3] is projected to grow significantly, with rTHA expected to rise by 19% by 2050 [4]. Despite advances in implant technology, surgical techniques, and biomaterials, rTHA presents significant challenges in the presence of severe acetabular bone defects. Traditional treatment options, such as impaction bone grafting coupled with various revision implants (e.g., hemispheric cups with hooks and flanges, iliac screw cups, or “cup in cage” constructs), often fall short in managing extensive acetabular bone defects classified as Paprosky Type IIIA, IIIB, or with pelvic discontinuity, due to high failure risks or feasibility issues [5,6,7,8,9,10]. The importance of accurate implant placement in these cases cannot be overstated, as it directly influences surgical outcomes and long-term implant stability. The emergence of additive manufacturing (3D printing) has enabled the creation of custom-made acetabular implants that are tailored to individual patient anatomy, offering a promising solution for severe acetabular defects [8,10,11,12,13]. These personalized implants are designed to precisely conform to the patient’s bone morphology, potentially enhancing the stability and alignment of hip joint reconstructions. Additionally, 3D printing allows for the incorporation of complex geometries and internal structures that are difficult to achieve with traditional manufacturing techniques. Therefore, additive manufacturing has promising potential in a wide range of orthopedic procedures [14]. However, there are also disadvantages to consider. The process can be time-consuming and expensive, particularly in the design and production phases. Furthermore, the long-term performance and durability of 3D-printed implants are still under investigation, with some studies indicating variability in mechanical properties and biocompatibility. Recent research has highlighted both the potential and the challenges of this technology in medical applications [14,15].

The precise placement of custom-made implants is critical for surgical success, requiring rigorous preoperative planning and execution. This includes using enhanced CT scans to accurately assess acetabular defects and design patient-specific implants [16]. Surgical outcomes are influenced by discrepancies between preoperative plans and actual intraoperative conditions, choice of surgical approach constrained by previous interventions, and the surgeon’s skill and experience. Furthermore, there remains a critical need to evaluate the accuracy of implant positioning postoperatively, which is essential for ensuring optimal patient outcomes. Postoperative imaging is crucial for verifying implant positioning to detect any deviations that could compromise implant longevity and patient outcomes. It provides valuable feedback for quality control and continuous improvement in surgical techniques. Additionally, it plays a pivotal role in follow-up assessments, monitoring implant integration, and identifying early signs of complications, with conventional 2D radiographs commonly used.

Recent literature highlights the importance of postoperative imaging, particularly 3D CT scans, for assessing the accuracy of implant placement. This assessment includes evaluating parameters such as the center of rotation (CoR), inclination (INCL), and anteversion (AV), and the contact between the implant and native bone, often yielding promising results [8,12,13,16,17,18]. Despite the superior accuracy of 3D CT scans, conventional 2D radiographs remain the gold standard in postoperative evaluations after rTHA due to their accessibility, cost-effectiveness, and lower radiation exposure. Given these advantages, using standard 2D radiographs to measure the positioning of custom-made acetabular implants offers a practical, less expensive alternative that could facilitate the implementation and quality control of these devices. However, the extent to which 2D radiographs can accurately evaluate the position of personalized acetabular components remains uncertain. The primary goal of this study was to assess whether 2D radiographs can serve as a viable alternative to 3D CT scans for postoperative evaluation of implant positioning. This study aimed to address this gap by evaluating the positioning of custom-made acetabular implants in a cohort of rTHA patients with severe acetabular bone defects (Paprosky ≥ Type IIIA) using either 3D CT scans or 2D radiographs compared to the positions planned preoperatively with CT imaging. To this end, we introduce a technique for aligning postoperative 2D X-rays with the CT-supported preoperative plans. Crucially, our findings suggest that conventional 2D radiographs are adequate for accurately assessing the placement of custom-made acetabular implants, thus offering valuable insights into the efficacy and feasibility of personalized implant solutions in complex hip revision surgeries.

## 2. Materials and Methods

### 2.1. Study Design

In this single-center, retrospective cohort study, we assessed the radiographic outcomes of patients who underwent revision hip surgery with custom-made acetabular implants at the Department of Orthopaedics, Heidelberg University Hospital. The surgeries were performed by two experienced surgeons, Tobias Reiner and Tilman Walker. Routine clinical imaging, including pre- and postoperative 3D CT scans and postoperative 2D plain radiographs, was used for the analysis, without additional specific scheduling for this study. Implant positions were measured using the CT and X-ray alignment methods, which are described in detail below, by two independent investigators. To evaluate interobserver variability, one investigator (Exp1) was a novice in measuring implant positions, while the other (Exp2) was seasoned in implant position analysis. In brief, the process of validating the assessment accuracy of 2D radiographs and 3D CT scans in evaluating the positioning of custom-made acetabular implants included the following steps: (1) Comparison of the postoperative implant positions obtained from 2D radiographs and 3D CT scans with the preoperative CT-based plans, (2) interobserver variability analysis to ensure the reliability of the measurements, (3) statistical analysis to determine whether there were significant differences between the measurements obtained from 2D radiographs and 3D CT scans, and (4) comparison of the observed deviations in implant positioning against clinically acceptable ranges established in the literature.

### 2.2. Patients

To establish and validate the X-ray alignment technique for assessing the implantation precision of custom-made acetabular implants, we identified seven patients who received these implants for large acetabular defects from January 2023 to October 2023. All patients had undergone rTHA and had postoperative 2D pelvic radiographs and 3D CT scans available. The selection for custom-made implantation was based on preoperative 3D CT scans identifying Paprosky Type III defects. Inclusion criteria required the availability of both postoperative imaging modalities. The reasons for acetabular revision included both aseptic and septic loosening. 

### 2.3. Custom-Made Acetabular Implants: Planning and Manufacturing

For surgical planning, a detailed evaluation using 3D CT scans was performed, covering the pelvic area and the existing hardware in the region of the hip joint and the knee and ankle joints. This comprehensive analysis assessed the condition of the acetabular component and quantified the bone loss severity. After obtaining informed consent, the 3D CT data were sent to AQ Solutions GmbH (Hürth, Germany) to produce the custom-tailored implant.

As previously noted [16], a semi-automatic bone segmentation algorithm was used to generate a 3D Computer-Aided Design (CAD) model of the pelvis, enhancing accuracy in reconstructing bone geometry—vital for cases with significant metallic components and radiation artifacts. The evaluation of the acetabular defect and the quality of the remaining bone informed the implant templating process, which included specifying screw orientation, iliac peg and flange placement, cup size and orientation, and areas for porous osseointegrative surface structures, customized for each patient’s needs.

Surgeons provided input on desired implant positioning, including anteversion, inclination, and careful restoration of the CoR. The CoR was determined based on physiological symmetry with the contralateral side, incorporating leg-length adjustments, and optimal acetabular cup positioning relative to patient-specific bone geometry, sometimes requiring minor adjustments for secure fixation. The final implant aimed to align within Lewinnek’s safe zone [19], targeting an inclination of 40° to 45° and an anteversion of 10° to 15°. The final implant, designed as monobloc system, was manufactured by powder bed fusion of a titanium alloy (Ti6Al4V) using a laser beam. Post-manufacturing steps included grinding, tapping, cleaning, washing, packaging, and sterilization, adhering to certified procedures of the legal manufacturer according to ISO 13485.

### 2.4. Surgical Procedure

Surgical interventions were conducted with patients in the lateral decubitus position using a lateral approach to the hip joint. The procedure began with the removal of the existing acetabular implant, followed by the extraction of any additional hardware and osteophytes as indicated by preoperative 3D CT scans. The custom-made acetabular implant was aligned according to the pre-determined plan and temporarily secured with Kirschner wires. Implant positioning was confirmed intraoperatively using fluoroscopy. Drill guides were used to accurately place a Kirschner wire for the central iliac peg, with its position verified fluoroscopically before utilizing a larger drill for final peg preparation and impaction. Additional implant fixation was achieved through the insertion of cup and flange screws, according to preoperative planning. Cavitary defects and voids were filled with allograft bone to ensure structural integrity. After securing the custom-made implant, a dual-mobility cup was cemented into place. Postoperatively, patients were instructed to initiate mobilization under partial weight-bearing conditions, with a load limit of 20 kg on the operated leg for the first 6 weeks. A 2D plain radiograph and 3D CT scan of the pelvis were taken postoperatively to verify proper implant placement and monitor for potential complications, such as periprosthetic fractures and secondary implant dislocation.

### 2.5. Quantitative Analysis of Implant Positioning Accuracy: 3D CT-Based Alignment

Postoperative 3D CT scans serve as the preferred imaging modality for assessing the precision of custom-made implant placement. As described elsewhere [16], we utilized a similar 3D approach to compare postoperative 3D CT data with the preoperative 3D CT template to evaluate implantation accuracy relative to preoperative planning. CT imaging was performed using high-resolution scanners (Siemens Healthineers, Somatom X.ceed, Erlangen, Germany) with a slice thickness of 2 mm, ensuring precise capture of bony structures with a pixel spacing of 0.478/0.478 mm and an image matrix of 512 × 512. The CT protocol included scanograms of the entire leg in both lateral and anterior-posterior views, followed by a three-scan series assessing the (1) pelvis (iliac crest—mid femur/below stem), (2) knee (condyles to calculate femoral rotation), and (3) ankle joints (to calculate the rotation of the tibia). Regular calibration and maintenance ensured consistent image quality.

Initially, an AI-driven semi-automatic method was used for the segmentation of the postoperative 3D CT model. To this end, a proprietary software from AQ Solutions GmbH was used, leveraging autonomous threshold-based segmentation followed by manual slice-by-slice refinement. Based on phantom validation, a slice accuracy below 2 mm was achieved. The AI-driven segmentation process is based on nnU-Net [20], a deep learning segmentation technique that self-adjusts for each new task, covering preprocessing, network architecture, training, and post-processing. The method achieved a dice score of 0.987 without hardware, indicating high accuracy.

**Figure 1:**
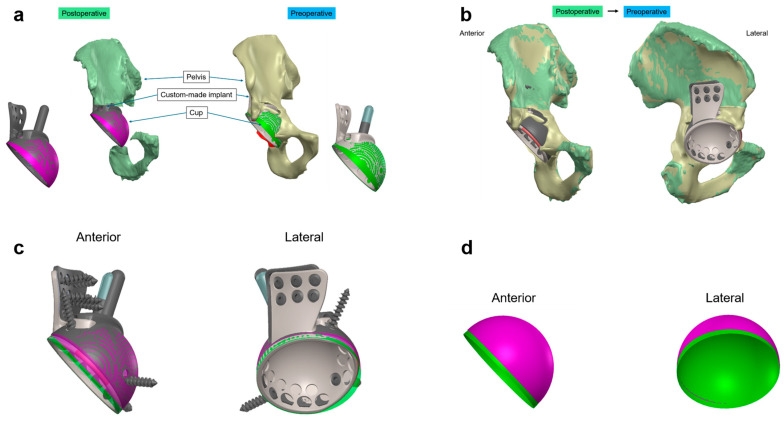
CT-based alignment strategy for custom-made acetabular implants (**a**) Visualization of the preoperative (beige) and postoperative (green) 3D pelvic models, showing the planned (green) and actual (purple) positions of the custom-made implant. (**b**) Overlay of the preoperative and postoperative CT scans illustrating the implant in situ within the surrounding bone structure. This comparison confirms the alignment accuracy between the pre- and postoperative data sets, achieving maximal bony overlay. (**c**) Extraction of the custom-made acetabular monobloc implant in anterior and lateral views, highlighting the differences between the planned preoperative (green) and actual postoperative (purple) positions. (**d**) Isolated view of the custom-made acetabular implant cup, illustrating deviations in the lateromedial, ventrodorsal, and craniocaudal directions, as well as anteversion and inclination angles in both anterior and lateral perspectives. Colors differentiate between preoperative (green) and postoperative (purple) conditions.

Then, the post- and preoperative 3D CT datasets were aligned using the Model Registration function of the open-source software 3D Slicer [21] (https://www.slicer.org; accessed on 29 May 2024), enabling an automated registration. This involved initial rough alignment based on anatomical landmarks and fine-tuning using an iterative closest point algorithm with a total of 1000 iterations, achieving a mean distance after registration consistently below 1 mm. Only the ala of the ilium and the caudal parts of the pubis and ilium were used for the registration to achieve a maximal bony overlay leaving out the acetabular region. The custom implant’s shape (monoblock) with its iliac peg served as implant references neglecting the screws. After ensuring the alignment between the pre- and postoperative CT datasets, deviations between the postoperative and planned implant positions were quantified with a proprietary specialized software from AQ Solutions using the hemispheric cup of the monobloc implant as the reference point for the entire implant’s position. This involved measuring the lateromedial (LM), ventrodorsal (VD), and craniocaudal (CC) translations, as well as anteversion (AV) and inclination (INCL). Measurements were conducted in discrete steps: angles were assessed in 0.1° increments, and center of rotation deviations (ML, VD, and CC directions) were evaluated in 0.1 mm steps. For visualization, the CAD program SolidWorks (Version 2019, Dassault Systèmes, Waltham, MA, USA) was used to display the deviations (Figure 1).

### 2.6. Quantitative Analysis of Implant Positioning Accuracy: 2D Radiograph-Based Alignment

**Figure 2:**
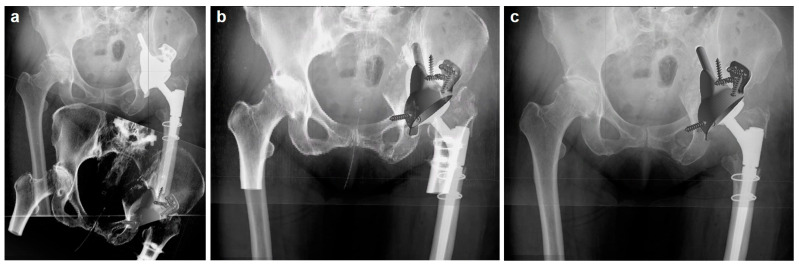
(**a**) Preoperative 3D CT-derived volume rendering of the pelvic bone with the planned implant position (bottom, gray implant), overlaid on the scaled 2D X-ray (top, white implant) to ensure dimensional accuracy. (**b**) Adjustment of the 3D CT volume-rendered pelvic model orientation to align with the X-ray, confirming anatomical landmark congruence while maintaining the implant’s planned position. (**c**) Final orientation adjustment with the pelvis fixed, showcasing the implant’s actual position in the X-ray for the postoperative analysis.

To quantitatively assess the accuracy of the implantation of the custom-made acetabular implants using 2D radiographs, we compared the postoperative anteroposterior X-rays of the pelvis with the preoperative 3D CT scans used for procedural planning and implant templating. Given the variation in patient posture and the complex geometry of the custom implants, proprietary specialized software from AQ Solutions was employed for the implant position assessment. This software also enabled the integration of 2D X-rays with overlaid 3D CT data. Initially, the 2D X-rays were imported into the software and scaled based on the specific dimensions of the implant. Subsequently, 3D models of the reconstructed pelvic bone, volume-rendered from the preoperative CT dataset and including the CAD implant model, were introduced (Figure 2a). The orientation of the volume-rendered pelvic model, along with the attached implant model, was then adjusted to align with the pelvic orientation in the X-rays. This adjustment ensured all anatomical landmarks aligned between the X-ray and the 3D pelvic model, while maintaining the implant in its planned position (Figure 2b). In the final step, the orientation of the pelvis was fixed, and only the implant orientation was altered to correspond with its actual position in the X-ray (Figure 2c). While matching 2D images to 3D models, a ventrodorsal translation of radiographs could not be entirely discounted. To address the inherent limitations of 2D X-rays and compensate for the absent third dimension, we considered physical boundaries such as bone geometry by reassessing the implant position and orientation using the preoperative transversal CT images. The final transformation (translation and rotation) was documented and recalculated to identify discrepancies in the CoR position, as well as variations in AV and INCL angles of the cup relative to the final implant specifications. All calculated parameters were benchmarked against the original CT dataset, disregarding any arbitrary pelvic tilting within the X-rays by employing the transformed CT reference system. Calculations were conducted in discrete steps: angles were assessed in 0.1° increments, and CC and LM positions were evaluated in 0.1 mm steps.

In alignment with established literature, malpositioning of the acetabular component was characterized by a deviation exceeding 10° for anteversion or inclination and a displacement greater than 5 mm from the intended CoR [12]. Additionally, the postoperative placement of the custom-made implant was assessed in the context of Lewinnek’s safe zone, which stipulates an optimal inclination range of 30° to 50° and an anteversion range of 5° to 25.

### 2.7. Statistical Analyses

All data are presented as mean ± standard deviation (SD) and include the range of minimum and maximum values where applicable. The distribution of data was evaluated using the D’Agostino–Pearson test to determine the appropriateness of parametric versus non-parametric statistical tests. Due to the non-normal distribution of most datasets, the Wilcoxon matched pairs signed-rank test was employed for comparing implantation parameters between preoperative 3D CT scans and postoperative imaging (both 2D X-ray and 3D CT), as well as for assessing differences between the measurements conducted by the two independent investigators (Exp1 vs. Exp2). The differences (Δ) in AV and INCL (measured in degrees) and the position of the CoR (defined in the CC, LM directions for X-ray, and additionally VD direction for 3D CT, measured in millimeters) were calculated by subtracting the preoperative CT values from the postoperative X-ray and CT values for each patient.

Statistical analyses were performed using GraphPad Prism version 9.5.1 for Windows (GraphPad Software, San Diego, CA, USA). The significance threshold (α) was set at 0.05, with *p*-values above this threshold denoted as not significant (ns).

## 3. Results

### 3.1. Study Population

The study cohort consisted of seven patients who received custom-made acetabular implants. The average age at implantation was 65.4 ± 8.0 years, ranging from 58 to 80 years. The majority of participants were female (5/7, 71%), and all (n = 7, 100%) underwent revision total hip arthroplasty (rTHA), primarily for aseptic loosening (6/7, 86%). Approximately half of these rTHA procedures (4/7, 57%) were performed as one-stage revisions. The interval between the preoperative 3D CT scan and surgery varied substantially from 37 to 289 days. Detailed patient demographics and clinical characteristics are outlined in Table 1.

### 3.2. Interobserver Variability Using 3D CT-Based Alignment

To assess the interobserver variability in analyzing the position of acetabular implants, two independent investigators (Exp1 and Exp2) measured implant positions using the 3D CT-based alignment technique. This method is the preferred imaging modality for assessing the precision of custom-made implant placement. The accuracy of the CoR reconstruction was evaluated by measuring translational changes between the planned preoperative and the actual postoperative implant positions in the craniocaudal (CC), lateromedial (LM), and ventrodorsal (VD) directions. The rotation of the personalized implant was assessed by measuring anteversion (AV) and inclination (INCL).

**Figure 3:**
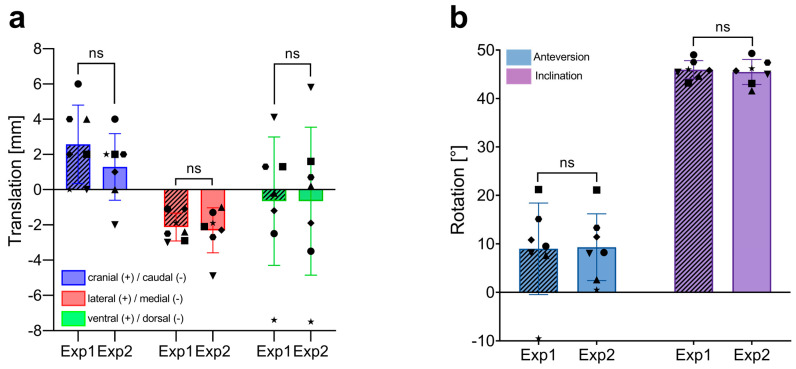
Interobserver variability in the measurement of implant positioning using 3D CT-based alignment. (**a**) Comparison of translational measurements (in mm) in the craniocaudal (CC), lateromedial (LM), and ventrodorsal (VD) directions between two independent investigators (Exp1, novice and Exp2, experienced). Each bar represents the mean ± SD, with individual data points showing the measurements of each patient (n = 7). (**b**) Comparison of rotational measurements (in degrees, °) for anteversion and inclination between the two investigators. Each bar represents the mean ± SD, with individual data points showing the measurements of each patient (n = 7). ns, not significant; SD, standard deviation.

There were no significant differences in CoR determination between Exp1 and Exp2 in any direction (Figure 3a). Exp1 recorded mean translations of 2.6 ± 2.2 mm (range: 0–6 mm) in CC, -2.1 ± 0.8 mm (range: −3–−1.1 mm) in LM, and −0.7 ± 3.6 mm (range: −7.4–4.1 mm) in VD. Exp2’s measurements were 1.3 ± 1.9 mm (range: −2–4 mm) in CC, −2.3 ± 1.3 mm (range: −4.8–−1.0 mm) in LM, and 0.7 ± 4.2 mm (range: −7.5–5.8 mm) in VD.

Both investigators also obtained similar results when analyzing the rotation of the implant, with mean AVs of 8.97 ± 9.4° (range: −9.5–21.2) for Exp1 and 9.3 ± 6.9° (range: 0.5–21.1) for Exp2. Mean INCL was 45.9 ± 1.9° (range: 43.2–49.0) for Exp1 and 45.5 ± 2.6° (range: 41.6–49.3) for Exp2. These findings indicate no significant difference in CoR and rotational measurements between a novice and an experienced investigator.

Combining the measurements from both investigators, the mean translational shifts between the preoperative templated and the actual postoperative implant positions were 1.9 mm, −2.2 mm, and −0.7 mm in the CC, LM, and VD directions, respectively, using the CT-based alignment strategy (as detailed in Table 2). These results indicate that the mean measurement error between the two independent investigators, using the gold standard CT-based method for implant position analysis, was up to 0.9 mm in the CC direction (as indicated by the SD). Standard deviations in the LM and VD directions were 0.4 mm and 0.3 mm, respectively.

The combined data from both investigators revealed average AV and INCL values of 9.1° and 45.7°, respectively, with deviations from the templated positions of −1.6° (ΔAV) and 1.4° (ΔINCL), as shown in Table 3. The measurement errors between the two investigators were minimal, with standard deviations of 1.4° for AV and 0.3° for INCL.

In summary, for 3D CT-based alignment, mean interobserver variability was up to 0.9 mm for translational shifts (CC direction) and 1.4° for rotation (AV).

### 3.3. Interobserver Variability Using 2D Radiograph-Based Alignment

**Figure 4:**
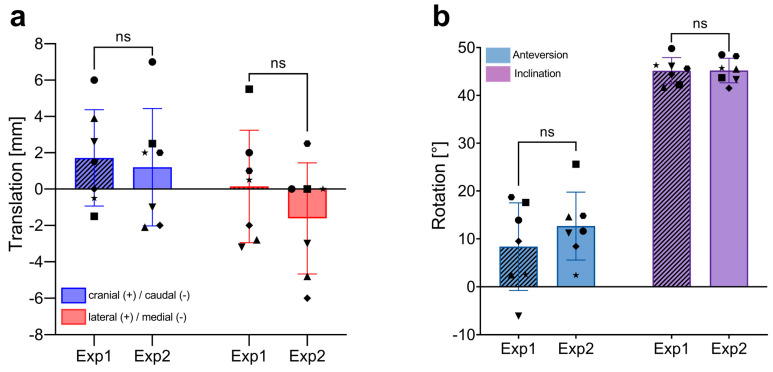
Interobserver variability in the measurement of implant positioning using 2D radiograph-based alignment. (**a**) Comparison of translational measurements (in mm) in the craniocaudal (CC), and lateromedial (LM) directions between two independent investigators (Exp1, novice and Exp2, experienced). Each bar represents the mean ± SD, with individual data points showing the measurements of each patient (n = 7). (**b**) Comparison of rotational measurements (in degrees, °) for anteversion and inclination between the two investigators. Each bar represents the mean ± SD, with individual data points showing the measurements of each patient (n = 7). ns, not significant; SD, standard deviation.

To evaluate the accuracy of 2D radiograph-based alignment in assessing implant placement compared to the commonly used 3D CT scan method, we first analyzed the interobserver variability of the 2D X-ray method as previously described. Similar to the CT-based analysis, the conventional 2D radiograph approach showed no significant differences between the two independent investigators in either the translational parameters for CoR reconstruction or the rotational aspects of the acetabular implants (Figure 4). The novice investigator (Exp1) recorded mean translational shifts of 1.7 ± 2.7 mm (range: −1.5–6) in the craniocaudal (CC) direction and 0.1 ± 3.1 mm (range: −3.2–5.5) in the lateromedial (LM) direction. The experienced investigator (Exp2) noted shifts of 1.2 ± 3.2 mm (range −2.1–7.0) in CC and −1.6 ± 3.1 mm (range −6.1–2.5) in LM. The ventrodorsal (VD) plane is not assessed in 2D radiograph alignment; thus, translational deviations in this direction are not analyzed.

Both investigators found similar rotational results, with mean anteversions (AV) of 8.4 ± 9.1° (range: −6.1–18.7) for Exp1 and 12.7 ± 7.1° (range: 2.4–25.6) for Exp2. Mean inclinations (INCL) were identical at 45.2° with a range of 41.7–49.8° for Exp1 and 41.5–48.5° for Exp2. These results demonstrate no significant difference in CoR and rotational measurements between a novice and an experienced investigator.

Combining the measurements from both investigators, the mean translational shifts between the preoperative templated and actual postoperative implant positions were 1.5 mm in the CC direction and −0.7 mm in the lateromedial (LM) direction, as per the 2D X-ray-based alignment strategy (detailed in Table 2). These findings indicate that the mean measurement error (standard deviation, SD) between the two independent investigators using the 2D radiograph-based method was up to 1.4 mm in the CC direction, with a standard deviation of 1.1 mm in the LM direction.

The combined data also showed average anteversion (AV) and inclination (INCL) values of 10.5° and 45.2°, respectively, with deviations from the templated positions of −0.2° (ΔAV) and 0.9° (ΔINCL) as indicated in Table 3. The measurement errors (SD) for rotational assessments were 3.2° for AV and 1.1° for INCL, reflecting greater variability compared to the CT-based method.

In summary, using the X-ray-based alignment strategy, mean interobserver variability was up to 1.4 mm for translational shifts (CC direction) and 3.2° for rotation (AV).

Overall, comparative analysis of the mean results from both investigators, encompassing all dimensions (CC, LM, AV, and INCL) for 2D and 3D matching, revealed no significant differences.

### 3.4. Outcome of Implant Rotation in Relation to Lewinnek’s Safe Zone

**Figure 5:**
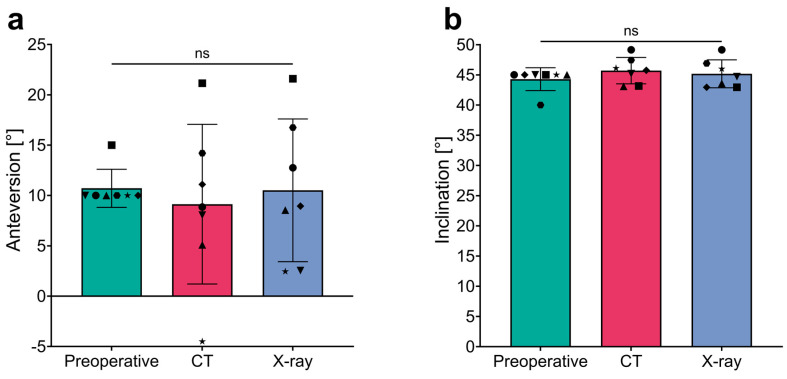
Comparison of anteversion and inclination measurements across different assessment techniques. (**a**) Anteversion and (**b**) inclination measurements in degrees (°) across three stages: Preoperative and postoperative assessments using the CT- and X-ray-based methods. The bar graph shows mean values with error bars representing standard deviations, and individual data points for each measurement technique. ns, not significant.

Preoperative templating defined absolute values for the planned anteversion (AV) and inclination (INCL) of the custom-made acetabular implants, allowing for both pre- and postoperative implant positions to be assessed relative to Lewinnek’s safe zone (AV: 5–25°; INCL: 30–50°). All personalized implants were templated to meet the safe zone criteria for AV and INCL. Specifically, AV was templated at 10° for six of the seven patients, with the remaining patient (Patient No. 3) having a planned AV of 15°, resulting in a planned mean AV of 10.7° (Table 3). Similarly, the mean planned INCL was 44.3°, with six implants templated at 45° and one (Patient No. 6) at 40°, all within the safe zone. Independent of the postoperative method (CT vs. X-Ray) used to analyze the final position of the implants, there were no significant differences in mean AV and INCL, neither between the two methods nor compared to the preoperative plan (Figure 5 and detailed in Table 3).

Upon detailed examination of individual data, deviations from Lewinnek’s safe zone for anteversion (AV; 5–25°) were observed. Specifically, using the CT-based alignment strategy, one patient (No. 2) presented an AV outside the safe zone with a value of −4.5° (Δ −14.5°). Similarly, with the X-ray method, two patients (No. 2 and No. 7) showed decreased AV values; Patient No. 2 recorded an AV of 2.45° (Δ −7.55°), and Patient No. 7 had an AV of 2.55°, both outside the safe zone. Notably, the CT-based assessment for Patient No. 7 reported an AV within the safe range at 8°. All seven patients demonstrated inclination (INCL) values within the safe range of 30–50°, regardless of the analysis technique used. Consequently, based on the analysis strategy employed, 85.7% (6/7) of patients evaluated with CT and 71.4% (5/7) with X-ray had final implant positions within the safe AV range proposed by Lewinnek. Inclination criteria were met by all patients across both the X-ray and CT modalities.

### 3.5. Accuracy Comparison of Implant Position Assessment between 3D CT and 2D Plane Radiograph-Based Alignment

**Figure 6:**
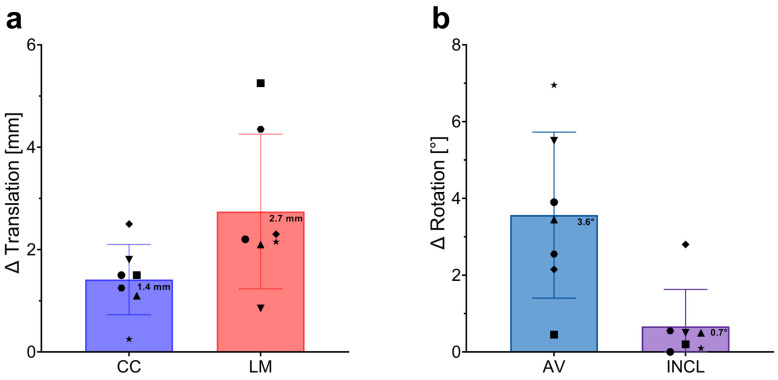
Differences in Implant Position Assessments Between 3D CT and 2D Plain Radiographs. (**a**) Differences in translational measurements (∆ Translation) in millimeters (mm) across the craniocaudal (CC) and lateromedial (LM) directions. The blue bar represents the average difference in the CC direction, while the red bar represents the average difference in the LM direction. Error bars indicate standard deviations, with individual data points representing the difference measurements for each patient. Mean values are clearly marked on each bar. (**b**) Differences in rotational measurements (∆ Rotation) in degrees (°) for anteversion (AV) and inclination (INCL). The blue bar shows the mean difference in AV, and the purple bar shows the mean difference in INCL. Error bars represent standard deviations, with individual data points for each patient displayed. Mean values are marked on the bars.

To evaluate the accuracy of conventional 2D plain radiographs in assessing the position of custom-made implants for large acetabular defects, we compared the results from 2D X-ray analysis with those from 3D CT analysis. Differences in translational measurements (Δ Translation) in the craniocaudal (CC) and lateromedial (LM) directions, as well as discrepancies in rotational measurements (anteversion, AV; and inclination, INCL), were calculated by subtracting the CT data from the X-ray data for each patient. The mean differences between the two assessment methods were 1.4 mm in the CC direction and 2.7 mm in the LM direction for the center of rotation (CoR) reconstruction. The minimal and maximal discrepancies in the CC direction were 0.3 mm and 2.5 mm, respectively, while in the LM direction, they were 0.9 mm and 5.3 mm, respectively. For implant rotation, we observed differences of 3.6° in AV and 0.7° in INCL when comparing CT with X-ray assessments. The minimal differences were 0.5° in AV and 0° in INCL, with maximal differences reaching 7.0° in AV and 2.8° in INCL (Figure 6).

In summary, these results demonstrate that assessing the position of custom-made acetabular implants using conventional 2D plain radiographs deviates from CT-based measurements by a maximum of 2.7 mm in the LM direction and 3.6° in AV, indicating a high level of accuracy for the 2D method.

## 4. Discussion

The primary objective of this study was to assess the accuracy of conventional postoperative 2D radiographs in evaluating the placement precision of personalized custom-made implants for significant acetabular bone defects (Paprosky ≥ IIIA Type). In this study we present an analysis technique that aligns postoperative X-rays with preoperative CT templates, enabling detailed assessment of the Center of Rotation (CoR) in two dimensions and implant rotation. These results were juxtaposed against the outcomes derived from postoperative 3D CT scans, which are considered the gold standard for evaluating the position of custom-made, patient-specific implants. Most notably, our findings reveal that conventional postoperative 2D plane radiographs provide sufficient accuracy for assessing the position of custom-made acetabular implants, challenging the prevailing necessity of more complex 3D imaging techniques.

To systematically validate the 2D radiograph-based alignment method, we first evaluated the accuracy of the gold standard—postoperative CT scans—to establish a reference for comparison. The interobserver variability was examined between an inexperienced and an experienced investigator. This approach was chosen to capture the maximal variability the method could present. Our analyses revealed a mean measurement error of up to 0.9 mm for CoR translation (CC direction) and a difference of 1.4° for implant rotation (AV). For LM translation and INCL, we found differences of 0.4 mm and 0.3°, respectively. These results illustrate the variability between two independent investigators using the same CT-based assessment technique for each dimension and serve as a reference for the X-ray-based assessment tool.

Similar to the 3D CT results, interobserver differences for CoR translations and implant rotation using the 2D radiograph-based method were most pronounced in the CC direction (1.4 mm) and AV (3.2°), suggesting that accurately determining craniocaudal CoR shifts and AV changes are more challenging compared to the lateromedial (LM) direction and INCL. The measurement errors between observers were slightly higher across all dimensions (CC, LM, AV, and INCL) when using conventional 2D radiographs for assessing implant positions compared to 3D CT-based assessments (Table 2 and Table 3).

To our best knowledge, only one study by Weber and colleagues has explored the comparative accuracy of 2D and 3D alignment strategies for assessing the positioning of custom-made acetabular implants [16]. They evaluated interobserver variability between two independent investigators using data from three cases and found agreement between observers with differences below 1 mm and 1° for 3D matching and 1 mm and 2° for 2D matching. With a maximal variability of 0.9 mm in CoR translation and 1.4° in rotation for 3D matching, our results align well with Weber’s findings. Similarly, the differences between observers that we observed for 2D matching (1.4 mm, 3.2°) are consistent with their results (1 mm, 2°). The slight differences between our findings and those of Weber’s study may be attributed to variations in the study populations and measurement specifics. While their study assessed three cases, our analysis explored interobserver variability with a larger sample size of seven. Additionally, specific details about the variability results across different dimensions, the measurement steps, and the experience levels of the investigators were not disclosed in Weber’s study, which could further explain the discrepancies observed.

Furthermore, we quantified the discrepancies between 2D and 3D assessments, revealing differences of 1.4 mm and 2.7 mm in the CC and LM directions for CoR analysis, and 3.6° and 0.7° for AV and INCL determination, respectively. Thus, analyzing the position of custom-made acetabular implants with 2D plane radiographs using 0.1 mm and 0.1° measurement steps provides results that deviate from the CT measurements by a mean of 2.7 mm in CoR translation and 3.6° in rotation. Considering that a comparative analysis of the mean results from both investigators across all dimensions (CC, LM, AV, and INCL) for 2D and 3D matching revealed no significant differences, this indicates that despite the greater relative variability observed in the measurements with the 2D method, both imaging techniques ultimately provide comparable levels of precision in assessing implant positions. Consistent with our findings, Weber and colleagues observed no significant deviations in inclination, LM, and CC positions between 2D and 3D assessments. However, they identified a significant difference in AV between the two methods [16]. Although in our study the AV differences (3.6°) between 2D and 3D matching did not reach statistical significance, they exhibited greater deviations compared to inclination (INCL) differences (0.7°). This is in line with findings from Bayraktar et al., who compared the accuracy of a commercially available radiographic planning software program with that of 3D-CT scans to analyze the cup rotation after THA. They reported that measurements of anteversion are particularly susceptible to errors, with mean inaccuracies of over 7°, in contrast to inclination inaccuracies (3.1°) [22].

In summary, our results indicate that conventional postoperative 2D radiographs provide largely accurate assessments of the position of custom-made acetabular implants. Considering the questionable clinical impact of the slight inaccuracies detected by X-rays, we contend that 2D radiographs are sufficient for evaluating the position of these implants. This holds particular importance in settings with limited resources where 3D imaging may not be readily available. Furthermore, the reduction in radiation exposure, cost, and the increased accessibility make 2D radiography a practical and attractive option for routine postoperative evaluation. Nonetheless, for detailed evaluations (such as checking the positioning of iliac pegs, detecting signs of loosening, etc.) and for research purposes, 3D CT scans continue to be the gold standard due to their unmatched accuracy and detail. Recent advancements in imaging technology, including biosensing and molecular imaging applications, have the potential to further improve accuracy assessment [23]. However, this potential has yet to be realized, as these technologies are still in the experimental stage.

Although not the primary focus of this study, our analysis of the final implant positions relative to the preoperative template yielded excellent results, regardless of whether 2D or 3D assessments were used. The 3D CT evaluations showed a mean cranial shift of 1.9 mm (X-ray: 1.5 mm) and a mean medial shift of 2.2 mm (X-ray: 0.7 mm) compared to the preoperatively planned CoR. These results are consistent with those from similar studies, which have reported mean cranial shifts ranging from 0.4 mm to 3.2 mm and individual craniocaudal deviations spanning from −6 mm to 18 mm [8,12,13,16,17,18]. Using 3D CT assessments, the majority of implants were positioned within a clinically acceptable deviation of less than 5 mm. However, one instance of malpositioning, involving a cranial deviation greater than 5 mm, was noted in one case (Patient No. 1; 1/7, 14.3%) when assessed using 2D radiographs. Additionally, the 3D CT analysis identified a dorsal CoR deviation exceeding 5 mm in another patient (Patient No. 2). Our rates of CoR malpositioning are comparable to those reported in other studies: Baauw et al. [12], Weber et al. [16], and Durand-Hill et al. [13] reported malpositioning rates of 18.75% (3/16), 9% (1/11), and 15% (3/20), respectively, in the CC dimension. These findings underscore the challenges inherent in accurately determining cup positions intraoperatively, a well-documented issue in both primary [24,25] and revision THA [16]. Woerner et al. investigated the reliability of intraoperative visual estimation for positioning the cup and stem in primary THA. They found that, even with significant surgical experience, visual assessment by the surgeon is not as accurate as 3D-CT imaging in minimally invasive THA procedures [24].

Following Lewinnek’s safe zone criteria (AV: 15° ± 10°; INCL: 40° ± 10°), our analysis found that 14.3% (1/7) of patients assessed with CT and 28.6% (2/7) assessed with X-ray had final implant positions outside the safe AV range. However, all patients met the inclination criteria across both X-ray and CT modalities. These findings align with those from similar studies. Weber et al. observed that 3 out of 11 implants (27.3%) were outside Lewinnek’s safe zone [16]. Baauw and colleagues reported deviations in 1 out of 16 cases (6.25%) for INCL and 2 out of 16 cases (12.5%) for AV [12]. Similarly, Zampelis and co-workers noted that 1 case (10%) and 2 cases (20%) out of 10 fell outside the safe zone for AV and INCL, respectively [17]. Freehand positioning of acetabular components in revision total hip arthroplasty (rTHA) is particularly challenging in the presence of extensive osseous defects. Choi and colleagues [26] noted that only 56% (19 out of 34) of implants in cases with Paprosky Type III defects were accurately placed within Lewinnek’s safe zone. Although Lewinnek’s criteria [19] are benchmarks for cup inclination and anteversion in primary THA, a significant number of THA dislocations occur even when components are positioned within these ranges [27]. Therefore, our primary goal during planning and templating was to ensure robust implant fixation, sometimes necessitating positions outside the safe zone due to compromised acetabular anatomy. This emphasizes the need for a personalized approach that prioritizes stable implant fixation over rigid adherence to conventional safe zones, particularly in complex cases requiring customized solutions. The placement of custom-made implants requires precise adaptation to each patient’s unique anatomical conditions, a task that becomes especially challenging in complex revision cases. These cases are often complicated by limited joint exposure, which can stem from extensive joint contracture, previous surgeries, and significant scar formation. The large-volume, custom-made partial pelvic replacements add to the complexity, as positioning these sizable implants accurately within a restricted joint space is daunting. Furthermore, potential bone loss during hardware removal and osteophyte clearance can compromise implant positioning. Moreover, the templating of custom-made 3D acetabular implants relies solely on osseous references, disregarding the influence of soft and scar tissues, which can significantly affect both the positioning and fixation of the implant. Together, these factors substantially increase the risk of implant malpositioning in patients with significant acetabular deficiencies [16]. A possible way to minimize the risk of implant malpositioning is the use of image-guided robotic-assisted solutions [28,29]. For joint arthroplasty, robots such as ROBODOC and MAKO use CT-based guidance and end-effector designs [28], which have demonstrated accurate implant positioning [29,30,31,32]. These systems leverage preoperative 3D CT scans to monitor implantation precision during surgery by registering anatomical landmarks, iteratively matched with the preoperative CT-based model [33,34]. Currently, the positioning of custom-made acetabular implants is conducted manually by orienting to visual and palpable anatomical landmarks identifiable during surgery, thus lacking intraoperative feedback on implantation precision. The integration of image-guided interventional robotics to position custom-made acetabular implants could represent a significant advancement in complex revision THA. However, robotic systems may require further development for more flexible registration of anatomical landmarks, especially in cases of severe acetabular defects. In such scenarios, predefined registration points necessary for real-time matching with the model might not exist or may be altered during surgery due to hardware removal or osteophyte resection. These points may need individual adjustment to match the patient’s specific anatomical conditions accurately. Despite these challenges, the integration of real-time monitoring through image-guided interventional robotics offers immediate feedback on the depth and orientation of the acetabular cup. This capability has the potential to significantly enhance the surgeon’s precision in implant placement. Nevertheless, future studies are necessary to confirm the feasibility and effectiveness of these procedures in revision THA, particularly with custom-made acetabular implants.

To further decrease the risk of implant malpositioning, enhancing congruency by reducing irregular large-size bone defects is crucial. Novel findings using polymers in medical implants might present several unique advantages, as reviewed by Xu et al. [35]. Polymers offer significant benefits in terms of biocompatibility [36], flexibility, and customizability [35]. They can mimic the mechanical properties of natural bone, reducing stress shielding and promoting better integration with host tissue [35]. Additionally, polymers can be enhanced with bioactive molecules [37] to support osteointegration [38] and reduce infection risk, which is particularly beneficial in revision hip arthroplasty where complications are more common. The versatility in manufacturing processes, such as 3D printing and injection molding, allows for creating complex geometries and patient-specific implants that closely match the defect site, improving fit and stability. Incorporating polymers or hybrid materials could potentially offer new avenues for improving implant performance and patient outcomes in revision hip arthroplasty.

Our study has several limitations. Its retrospective design and relatively small sample size may limit the generalizability of our findings. Moreover, due the small study population, subgroup analyses (e.g., cause of loosening—aseptic/septic—and type of revision surgery—one-/two-stage) could not be performed. These factors need to be addressed in future studies, as they might influence implant position and functional outcomes. Therefore, larger sample sizes, ideally from multicentric prospective trials, are needed to corroborate our results and address these critical unresolved issues. To minimize selection bias, we included all patients who received custom-made partial pelvic replacements for large acetabular defects (≥Paprosky Type IIIA) and who had both postoperative 2D and 3D images available. We gathered data from seven patients over a period of less than 10 months (01/2023–10/2023). This sample size is considerable given that benchmark studies in this field typically report cohorts ranging from 3 to 45 patients collected over several years. Nonetheless, the absence of long-term follow-up data limits our ability to evaluate the lasting effects of our findings on implant longevity and function. Specifically, patient-reported and functional outcome measurements need to be included and related to the implant position in future studies. It has already been shown that treatment outcomes are significantly influenced by the accuracy and reliability of imaging technologies used in the treatment [39].

Additionally, the uniform surgical approach in our study might introduce bias; all operations were conducted in the lateral decubitus position using a lateral approach to the hip joint. The process of matching 2D images to 3D models also presents inherent methodological challenges, notably in quantifying shifts in the ventrodorsal direction with precision using 2D radiographs. Although the unique geometry of the implant and the presence of metallic augmentation and screw fixation suggest that a VD shift would impact other spatial parameters, this does not provide a direct measure of VD translation. To address this, we cross-referenced adjusted implant positions and orientations with the preoperative transversal CT plane, utilizing CT’s capacity to provide a three-dimensional perspective to ensure accuracy.

Our method allowed us to assess angles and translational shifts in 0.1° and 0.1-mm increments with 2D radiographs, offering precision comparable to 3D CT scans, which is clinically excellent and sufficient. However, given the higher measurement errors across all dimensions with the X-ray method, 3D CT scans still provide more precise localization of the implant and remain superior for exact spatial determinations.

To capture the maximal variability of the method, we intentionally included both an inexperienced and an experienced investigator, acknowledging that interobserver variability could have been further minimized with more cases and additional observers. Intraobserver variability was not assessed to prevent a learning effect for the inexperienced investigator.

Future research involving larger cohorts and extended follow-up periods that include clinical evaluations is crucial to validate our results and fully understand the long-term clinical implications of the precision of implant positioning as determined by 2D radiography.

## 5. Conclusions

In conclusion, the current study demonstrates the potential of using conventional postoperative 2D radiographs to accurately assess the placement of custom-made acetabular implants in revision total hip arthroplasty for severe acetabular defects. Our findings indicate that 2D radiographs offer a viable alternative that can sufficiently measure implant positioning. These results support the use of 2D radiography for routine postoperative evaluation, while 3D CT scans remain superior for detailed assessments.

## Figures and Tables

**Table 1:** Study population: patient characteristics.

Study Population	
**Age, years ± SD (range)**	65.4 ± 8.0 (58–80)
Sex, n (%)	
Female	5 (71.4)
Male	2 (28.6)
**BMI, kg/m^2^ ± SD (range)**	26.9 ± 6.8 (20.3–32.9)
**Hip, n (%)**	
Right	3 (42.9)
Left	4 (57.1)
**Surgery, n (%)**	
Revision	7 (100)
One-stage	4 (57.1)
Two-stage	3 (42.9)
**Cause, n (%)**	
Septic loosening	1 (14.3)
Aseptic loosening	6 (85.7)
**Time (CT—surgery), days ± SD (range)**	145 ± 85.3 (37–289)

BMI, Body Mass Index; n, number of patients; SD, standard deviation.

**Table 2:** Mean translation measurements of implant positioning using 3D CT and 2D radiographs.

Translation										
	3D CT						2D Radiographs			
	Cranial (+)/Caudal (−) [mm]		Lateral (+)/Medial (−) [mm]		Ventral (+)/Dorsal (−) [mm]		Cranial (+)/Caudal (−) [mm]		Lateral (+)/Medial (−) [mm]	
Patient No.	Mean	SD	Mean	SD	Mean	SD	Mean	SD	Mean	SD
1	5	1	−1.2	0.1	−3	0.5	6.5	0.5	1	1
2	1	1	−1.9	0	−7.45	0.05	0.75	1.25	0.25	0.25
3	2	0	−2.5	0.4	1.45	0.15	0.5	2	2.75	2.75
4	2	2	−1.7	0.7	0	0.2	0.9	3	−3.8	1
5	1.5	0.5	−1.7	0.6	−1.55	0.35	−1	1	−4	2
6	3	1	−2.6	0.1	1	0.3	1.75	0.25	1.75	0.75
7	−1	1	−3.95	0.95	4.95	0.85	0.8	1.8	−3.1	0.1
Min.	−1.0	0.0	−4.0	0.0	−7.5	0.0	−1.0	0.3	−4.0	0.1
Mean	1.9	0.9	−2.2	0.4	−0.7	0.3	1.5	1.4	−0.7	1.1
Max.	5.0	2.0	−1.2	1.0	5.0	0.9	6.5	3.0	2.8	2.8

Max., maximum; Min., minimum; SD, standard deviation.

**Table 3:** Mean rotational measurements of implant positioning using 3D CT and 2D radiographs.

Rotation														
	Preoperative Plan		3D CT						2D Radiographs					
Patient No.	AV Plan [°]	INCL Plan [°]	AV Final [°]	SD	ΔAV [°]	INCL Final [°]	SD	ΔINCL [°]	AV Final [°]	SD	ΔAV [°]	INCL Final [°]	SD	ΔINCL [°]
1	10	45	8.85	0.65	−1.15	49.15	0.15	4.15	12.75	1.15	2.75	49.15	0.65	4.15
2	10	45	−4.5	5	−14.5	46.1	0.1	1.1	2.45	0.05	−7.55	46	0.3	1
3	15	45	21.15	0.05	6.15	43.15	0.05	−1.85	21.6	4	6.6	42.95	0.75	−2.05
4	10	45	5.1	2.5	−4.9	43.1	1.5	−1.9	8.55	6.05	−1.45	43.6	1.9	−1.4
5	10	45	11.1	0.3	1.1	45.75	0.05	0.75	8.95	0.55	−1.05	42.95	1.45	−2.05
6	10	40	14.2	0.9	4.2	47.45	0.05	7.45	16.75	1.95	6.75	46.9	1.3	6.9
7	10	45	8.05	0.05	−1.95	45.2	0.2	0.2	2.55	8.65	−7.45	44.7	1.4	−0.3
Min.	10	40	−4.5	0.0	−14.5	43.1	0.0	−1.9	2.5	0.1	−7.6	43.0	0.3	−2.1
Mean	10.7	44.3	9.1	1.4	−1.6	45.7	0.3	1.4	10.5	3.2	−0.2	45.2	1.1	0.9
Max.	15	45	21.2	5.0	6.2	49.2	1.5	7.5	21.6	8.7	6.8	49.2	1.9	6.9

AV, anteversion; INCL, inclination; SD, standard deviation.

## Data Availability

All data supporting the findings of this study are available within the article. Raw data are available on request from the corresponding author.

## References

[1] Learmonth I.D., Young C., Rorabeck C. (2007). The operation of the century: Total hip replacement. Lancet.

[2] Shichman I., Roof M., Askew N., Nherera L., Rozell J.C., Seyler T.M., Schwarzkopf R. (2023). Projections and Epidemiology of Primary Hip and Knee Arthroplasty in Medicare Patients to 2040–2060. JBJS Open Access.

[3] Labek G., Thaler M., Janda W., Agreiter M., Stöckl B. (2011). Revision rates after total joint replacement. J. Bone Jt. Surg. Br. Vol..

[4] Rasmussen M.B., El-Galaly A., Daugberg L., Nielsen P.T., Jakobsen T. (2022). Projection of primary and revision hip arthroplasty surgery in Denmark from 2020 to 2050. Acta Orthop..

[5] Beswick A., Blom A.W. (2011). Bone graft substitutes in hip revision surgery: A comprehensive overview. Injury.

[6] Zhang Y., Gao Z., Zhang B., Du Y., Ma H., Tang Y., Liu Y., Zhou Y. (2022). The application of custom-made 3D-printed titanium augments designed through surgical simulation for severe bone defects in complex revision total hip arthroplasty. J. Orthop. Traumatol..

[7] Yeroushalmi D., Singh V., Maher N., Gabor J.A., Zuckerman J.D., Schwarzkopf R. (2023). Excellent mid-term outcomes with a hemispheric titanium porous-coated acetabular component for total hip arthroplasty: 7–10 year follow-up. HIP Int..

[8] Romagnoli M., Zaffagnini M., Carillo E., Raggi F., Casali M., Leardini A., Marcheggiani Muccioli G.M., Grassi A., Zaffagnini S. (2023). Custom-made implants for massive acetabular bone loss: Accuracy with CT assessment. J. Orthop. Surg. Res..

[9] Cadossi M., Garcia F.L., Sambri A., Andreoli I., Dallari D., Pignatti G. (2017). A 2- to 7-Year Follow-Up of a Modular Iliac Screw Cup in Major Acetabular Defects: Clinical, Radiographic and Survivorship Analysis With Comparison to the Literature. J. Arthroplast..

[10] Baauw M., van Hooff M.L., Spruit M. (2016). Current Construct Options for Revision of Large Acetabular Defects: A Systematic Review. JBJS Rev..

[11] Meding J.B., Meding L.K. (2023). Custom Triflange Acetabular Implants: Average 10-Year Follow-Up. J. Arthroplast..

[12] Baauw M., Hellemondt G.G.v., Hooff M.L.v., Spruit M. (2015). The accuracy of positioning of a custom-made implant within a large acetabular defect at revision arthroplasty of the hip. Bone Jt. J..

[13] Durand-Hill M., Henckel J., Di Laura A., Hart A.J. (2020). Can custom 3D printed implants successfully reconstruct massive acetabular defects? A 3D-CT assessment. J. Orthop. Res..

[14] Qu Z., Yue J., Song N., Li S. (2024). Innovations in 3D printed individualized bone prosthesis materials: Revolutionizing orthopedic surgery: A review. Int. J. Surg..

[15] Anzillotti G., Guazzoni E., Conte P., Di Matteo V., Kon E., Grappiolo G., Loppini M. (2024). Using Three-Dimensional Printing Technology to Solve Complex Primary Total Hip Arthroplasty Cases: Do We Really Need Custom-Made Guides and Templates? A Critical Systematic Review on the Available Evidence. J. Clin. Med..

[16] Weber M., Witzmann L., Wieding J., Grifka J., Renkawitz T., Craiovan B. (2019). Customized implants for acetabular Paprosky III defects may be positioned with high accuracy in revision hip arthroplasty. Int. Orthop..

[17] Zampelis V., Flivik G. (2020). Custom-made 3D-printed cup-cage implants for complex acetabular revisions: Evaluation of pre-planned versus achieved positioning and 1-year migration data in 10 patients. Acta Orthop..

[18] Wessling M., Gebert C., Hakenes T., Dudda M., Hardes J., Frieler S., Jeys L.M., Hanusrichter Y. (2022). Reconstruction of Paprosky III defects with custom-made implants: Do we get them in the correct position?. Bone Jt. J..

[19] Lewinnek G.E., Lewis J., Tarr R., Compere C., Zimmerman J. (1978). Dislocations after total hip-replacement arthroplasties. JBJS.

[20] Isensee F., Jaeger P.F., Kohl S.A.A., Petersen J., Maier-Hein K.H. (2021). nnU-Net: A self-configuring method for deep learning-based biomedical image segmentation. Nat Methods.

[21] Fedorov A., Beichel R., Kalpathy-Cramer J., Finet J., Fillion-Robin J.C., Pujol S., Bauer C., Jennings D., Fennessy F., Sonka M. (2012). 3D Slicer as an image computing platform for the Quantitative Imaging Network. Magn. Reason. Imaging.

[22] Bayraktar V., Weber M., von Kunow F., Zeman F., Craiovan B., Renkawitz T., Grifka J., Woerner M. (2017). Accuracy of measuring acetabular cup position after total hip arthroplasty: Comparison between a radiographic planning software and three-dimensional computed tomography. Int. Orthop..

[23] Jiang Z., Han X., Zhao C., Wang S., Tang X. (2022). Recent Advance in Biological Responsive Nanomaterials for Biosensing and Molecular Imaging Application. Int. J. Mol. Sci..

[24] Woerner M., Sendtner E., Springorum R., Craiovan B., Worlicek M., Renkawitz T., Grifka J., Weber M. (2016). Visual intraoperative estimation of cup and stem position is not reliable in minimally invasive hip arthroplasty. Acta Orthop..

[25] Barrack R.L., Krempec J.A., Clohisy J.C., McDonald D.J., Ricci W.M., Ruh E.L., Nunley R.M. (2013). Accuracy of Acetabular Component Position in Hip Arthroplasty. JBJS.

[26] Choi H.R., Anderson D., Foster S., Beal M., Lee J.A., Barr C., Malchau H., McCarthy J., Kwon Y.M. (2013). Acetabular cup positioning in revision total hip arthroplasty with Paprosky type III acetabular defects: Martell radiographic analysis. Int. Orthop..

[27] Abdel M.P., von Roth P., Jennings M.T., Hanssen A.D., Pagnano M.W. (2016). What Safe Zone? The Vast Majority of Dislocated THAs Are Within the Lewinnek Safe Zone for Acetabular Component Position. Clin. Orthop. Relat. Res..

[28] Fichtinger G., Troccaz J., Haidegger T. (2022). Image-guided interventional robotics: Lost in translation?. Proc. IEEE.

[29] Kim K., Kwon S., Kwon J., Hwang J. (2023). A review of robotic-assisted total hip arthroplasty. Biomed. Eng. Lett..

[30] Nawabi D.H., Conditt M.A., Ranawat A.S., Dunbar N.J., Jones J., Banks S., Padgett D.E. (2013). Haptically guided robotic technology in total hip arthroplasty: A cadaveric investigation. Proc. Inst. Mech. Eng. Part H J. Eng. Med..

[31] Elson L., Dounchis J., Illgen R., Marchand R.C., Padgett D.E., Bragdon C.R., Malchau H. (2015). Precision of acetabular cup placement in robotic integrated total hip arthroplasty. Hip Int..

[32] Domb B.G., El Bitar Y.F., Sadik A.Y., Stake C.E., Botser I.B. (2014). Comparison of robotic-assisted and conventional acetabular cup placement in THA: A matched-pair controlled study. Clin. Orthop. Relat. Res..

[33] Nakamura N., Sugano N., Nishii T., Miki H., Kakimoto A., Yamamura M. (2009). Robot-assisted primary cementless total hip arthroplasty using surface registration techniques: A short-term clinical report. Int. J. Comput. Assist. Radiol. Surg..

[34] Tarwala R., Dorr L.D. (2011). Robotic assisted total hip arthroplasty using the MAKO platform. Curr. Rev. Musculoskelet. Med..

[35] Xu Y., Zhang F., Zhai W., Cheng S., Li J., Wang Y. (2022). Unraveling of Advances in 3D-Printed Polymer-Based Bone Scaffolds. Polymers.

[36] Dai W., Sun M., Leng X., Hu X., Ao Y. (2020). Recent Progress in 3D Printing of Elastic and High-Strength Hydrogels for the Treatment of Osteochondral and Cartilage Diseases. Front. Bioeng. Biotechnol..

[37] Zhang W., Feng C., Yang G., Li G., Ding X., Wang S., Dou Y., Zhang Z., Chang J., Wu C. (2017). 3D-printed scaffolds with synergistic effect of hollow-pipe structure and bioactive ions for vascularized bone regeneration. Biomaterials.

[38] He Y., Wang W., Tang X., Liu X. (2017). Osteogenic induction of bone marrow mesenchymal cells on electrospun polycaprolactone/chitosan nanofibrous membrane. Dent. Mater. J..

[39] Hu J., Xu T., Shen H., Song Y., Yang J., Zhang A., Ding H., Xing N., Li Z., Qiu L. (2023). Accuracy of Gallium-68 Pentixafor Positron Emission Tomography–Computed Tomography for Subtyping Diagnosis of Primary Aldosteronism. JAMA Netw. Open.

